# Psychotherapy program as an additional treatment method for patients with rheumatoid arthritis, multidisciplinary approach

**DOI:** 10.1192/j.eurpsy.2023.2173

**Published:** 2023-07-19

**Authors:** A. Matic, A. Gudelj Gračanin, I. Tonković, L. Mužinić Marinić, J. Morović-Vergles

**Affiliations:** 1 Department of Psychotherapy, University Psychiatric Hospital Vrapče; 2 Department of Clinical Immunology, Rheumatology and Pulmonology, Clinical Hospital “Sv. Duh”; 3 retired; 4 Department of psychiatry; 5 Department of Immunology, Allergology and Rheumatology, University Hospital Dubrava, Zagreb, Croatia

## Abstract

**Introduction:**

Chronic diseases, with the development of medicine, the extension of life expectancy and the reduction of dying from infectious diseases, have become a leading public health problem. Rheumatoid arthritis (RA) is a chronic, progressive, multifactorial, autoimmune, systemic inflammatory disease of connective tissue, and sufferers are at greater risk of developing psychological disorders, depression and anxiety being the most common. Optimal treatment of RA should include periodic screening of somatic and psychological comorbid disorders and diseases with a multidisciplinary approach to treatment.

**Objectives:**

Presenting a psychotherapy program as an additional method of treating patients with rheumatoid arthritis.

**Methods:**

In University Hospital Dubrava, a psychotherapy program was organized for patients with rheumatoid arthritis, which took place once a week for 12 weeks, and consisted of autogenic training, education about the disease and group psychotherapy. The program would begin with autogenic training held by a clinical psychologist, and the main goal was to achieve psychophysical relaxation. This was followed by an education on rheumatoid arthritis, in which a clinical rheumatologist was involved, and the goal was to provide basic information about the disease, thereby achieving better cooperation with health care personnel and a more active role in their own treatment. In the end, there would be a time-limited dynamic group psychotherapy conducted by a psychiatrist/psychotherapist with the basic therapeutic effects of group therapy.

**Results:**

As a result of a 12-week psychotherapy program for somatic patients, which was held in a clinical hospital, patients’ anxiety and depression decreased, the experience of pain decreased, and there were positive changes in the perception of the quality of life.

**Conclusions:**

A multidisciplinary approach is recommended in treating patients with rheumatoid arthritis and psychotherapy progam can be used as an additonal way of treatment.

**Disclosure of Interest:**

None Declared

